# Protein Stability, Folding and Misfolding in Human PGK1 Deficiency

**DOI:** 10.3390/biom3041030

**Published:** 2013-12-18

**Authors:** Giovanna Valentini, Maristella Maggi, Angel L. Pey

**Affiliations:** 1 Dipartimento di Biologia e Biotecnologie “L. Spallanzani”, Università degli Studi di Pavia, Viale Taramelli, 3B, Pavia 27100, Italy; E-Mail: giovanna.valentini@unipv.it (G.V.); maristella.maggi01@universitadipavia.it (M.M.); 2 Department of Physical Chemistry, Faculty of Science, University of Granada, Av. Fuentenueva s/n, Granada 18071, Spain

**Keywords:** protein misfolding, protein aggregation, conformational disease, pharmacological therapies, molecular chaperones, thermodynamic stability, kinetic stability, proteolysis

## Abstract

Conformational diseases are often caused by mutations, altering protein folding and stability *in vivo*. We review here our recent work on the effects of mutations on the human phosphoglycerate kinase 1 (hPGK1), with a particular focus on thermodynamics and kinetics of protein folding and misfolding. Expression analyses and *in vitro* biophysical studies indicate that disease-causing mutations enhance protein aggregation propensity. We found a strong correlation among protein aggregation propensity, thermodynamic stability, cooperativity and dynamics. Comparison of folding and unfolding properties with previous reports in PGKs from other species suggests that hPGK1 is very sensitive to mutations leading to enhance protein aggregation through changes in protein folding cooperativity and the structure of the relevant denaturation transition state for aggregation. Overall, we provide a mechanistic framework for protein misfolding of hPGK1, which is insightful to develop new therapeutic strategies aimed to target native state stability and foldability in hPGK1 deficient patients.

## 1. Introduction

Protein folding inside eukaryotic cells is a complex process wherein folding of the newly synthesized polypeptide competes with aggregation and degradation [1]. Macromolecular crowding and excluded volume effects may enhance intermolecular associations intracellularly, thus increasing the aggregation propensity of partially folded states [1]. Nature has provided living organisms with a complex network of proteins responsible for efficient protein folding, trafficking and degradation, collectively known as the protein homeostasis network [2,3,4,5]. However, mutations in human genes may cause conformational diseases by altering protein foldability (*i.e.* protein homeostasis). These mutations may affect physical properties of the protein (such as thermodynamic stability, folding/unfolding/misfolding rates) or the interaction of the protein along its folding process with elements of the protein homeostasis network [2,3,4,5]. In the case of conformational diseases with protein loss-of-function, alterations in protein homeostasis due to mutations may cause a reduction in a certain enzymatic function, leading to metabolic alterations (*i.e.* metabolic inherited diseases). Current therapies for inherited metabolic diseases usually rely on dietary restrictions to avoid accumulation of toxic metabolites. Dietary therapies often work only for a fraction of patients prolonging their life span and quality of life, and these therapies are socially and economically burdening. In many cases, the patients may benefit from pharmacological supplementation with vitamins and protein cofactors, which may work through alleviation of protein folding and stability defects [6,7]. Alternative therapies targeting specific alteration in protein homeostasis, using pharmacological chaperones that target the folding and stability of the protein, or protein homeostasis modulators which boost or fine-tune the protein homeostasis capacity to cope with folding defects, are promising approaches to treat conformational diseases [6,7,8]. Development of these pharmacological therapies often require insight on the effects of mutations on protein homeostasis to identify those defects that must be targeted by pharmacological ligands. 

**Figure 1:**
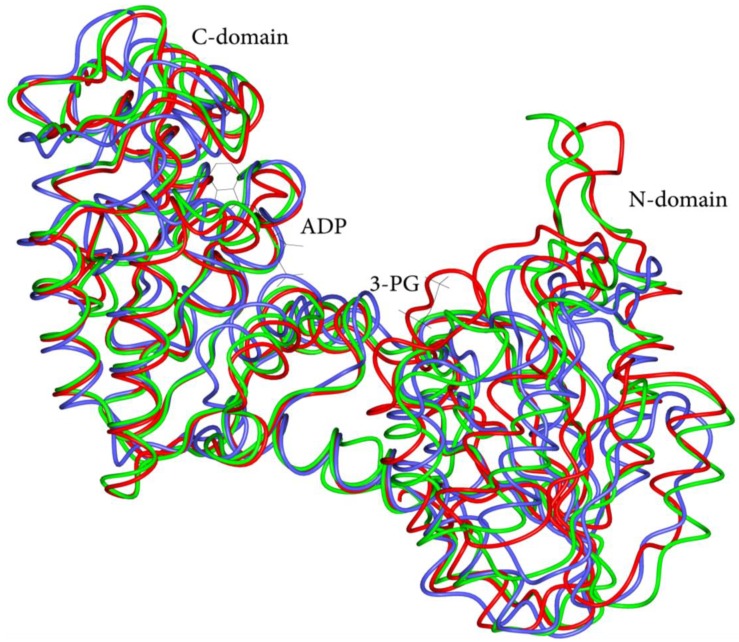
Superimposition of the C^α^ traces of three PGK structures: *Escherichia coli* (blue, PDB ID 1ZMR), *Saccharomyces cerevisiae* (green, PDB ID 1FW8) and *Homo sapiens* (red, PDB ID 2XE7). The conformation of each individual domain, except for a few source-specific insertions, is similar in all structures. Both inter-domain helix and L hinge are almost completely superposed in all the three PGKs, pointing to a conservative evolution of the structural elements and underlining their essential role in the protein function. The bound ligands are shown as thin stick models.

In this review, we describe our recent efforts to understand the impact of mutations in folding and stability of the human phosphoglycerate kinase 1 (hPGK1) enzyme, which are associated to hPGK1 deficiency, a conformational disease. PGK is an essential enzyme for all living organisms that catalyzes the reversible phosphotransfer reaction from 1,3-bisphosphoglycerate (1,3-BPG) to ADP to form ATP and 3-phosphoglycerate (3-PG) [9]. Two isoforms of PGK exists in humans (namely hPGK1 and hPGK2), which are functionally and structurally alike [10,11,12]. The three-dimensional fold of PGK is highly conserved among prokaryotic and eukaryotic enzymes, displaying an archetypical two-domain structure ([12] and Figure 1). 

PGK enzymes have been studied extensively as a model system for the folding/unfolding of two-domains proteins, including bacterial, yeast and mammalian PGKs [13,14,15,16,17,18,19,20]. Despite their size and structural complexity, the unfolding of PGK enzymes is often described well by relatively simple models. Equilibrium unfolding of human, *E. coli* and yeast PGKs follows a simple two-state equilibrium model, yielding denaturation free energies in the range of 8–10 kcal·mol^−1^ [13,15]. However, the denaturation equilibrium of some PGK enzymes seems to be more complex, with a significant population of equilibrium unfolding intermediates [14]. Kinetic folding/unfolding studies have also supported a remarkably complex behavior in some PGK enzymes, with the population of kinetic intermediates [21,22,23]. The dynamics of the native state ensemble have also been addressed in several works, showing large differences in protein dynamics between PGK enzymes from different organisms [13,15]. Interestingly, studies inside living cells and in the presence of macromolecular crowding agents have shown that physical and functional properties of PGK enzymes, including folding kinetics and stability, as well as the domain dynamics undergone along the catalytic cycle, may be strongly affected by macromolecular crowding *in vivo* [24,25,26]. All these studies also shed light on the changes in protein denaturation energetics and folding/unfolding cooperativity in an evolutionary perspective. 

## 2. Human PGK1 Deficiency

### 2.1. Clinical Presentation and Current Treatments

hPGK1 deficiency (OMIM ID 311800) is a rare genetic disease, documented in approximately 40 unrelated kindreds [9,27,28,29]. It is characterized by pleomorphic clinical presentation including chronic non-spherocytic hemolytic anemia, neurological disfunctions and myopathy. Rarely, patients exhibit all three clinical features of hPGK1 deficiency [9,27,30]. hPGK1 deficient patients generally show hemolytic anemia in association with neurological manifestations or myopathy with or without central nervous system (CNS) involvement. The hemolytic anemia is mainly of mild to severe degree. Marked exacerbations in the disorder often occur during infections [31]. Myopathy is a progressive disease, characterized by exercise intolerance, muscle weakness, cramping, myalgia and episodes of myoglobinuria during strenuous exercise or fever [32,33,34,35]. Neurological manifestations include mental retardation, progressive decline of motor function, developmental delay, seizures, epilepsy, ataxia, tremor, hemiplegic migraines, retinopathy, progressive dysphagia for solid and liquids [36,37,38]. hPGK1 deficiency is inherited as an X -linked recessive trait. Thus, males have full expression of the disorder, whereas females are usually asymptomatic with a population of deficient cells coexisting with a normal cell population. Even though few cases have been reported, hPGK1 deficiency has a world-wide distribution [9]. As for the disease treatment, no specific therapy for hPGK1 deficiency is available. Red cell transfusions may be required in severely anemic cases, particularly in the first years of life or during intercurrent infections or other conditions. Splenectomy has a favorable outcome in some cases, but does not correct the hemolytic process [9,39]. Recently, an allogeneic bone marrow transplant for hPGK1 deficiency has been attempted to arrest neurological manifestations development [9,37,38,40,41]. 

### 2.2. Genetics and Genotype/Phenotype Correlations in hPGK1 Deficiency

Two functional loci for the production of PGK have been identified in the mammalian genome. The human *PGK-1* gene is constitutively expressed in all somatic cells and premeiotic cells, mapping to chromosome Xq13.3. It spans nearly 23 kilobases, and consists of 11 exons and 10 introns [10,42]. The *PGK-2* gene is expressed in a tissue specific manner in meiotic/postmeiotic spermatogenic cells, mapping to chromosome 6p12-21.1, it is intronless and displays the characteristics of a retroposon [11]. Both hPGK1 and hPGK2 isoenzymes are 417 amino acid-long with a molecular mass of approximately 45 kDa.

Mutations in the *PGK-1* gene result in hPGK1 deficiency (OMIM ID 311800). Since the first description [43,44], nearly 40 cases have been reported, and 28 of them characterized at DNA or protein level. Among the 21 different mutations identified in *PGK-1* gene, sixteen cause amino acid substitutions, two are deletions of the coding region and three alterations of the splicing site (Table 1). Mutations are spread all along the gene and are puzzlingly associated to different phenotypes. Recent studies performed on hPGK1 mutant enzymes allowed highlight the effects of *PGK-1* gene mutations on the enzyme, correlating the molecular alterations to the different pathological manifestations [13,27,45,46]. 

## 3. Functional and Stability Defects in Human PGK1 Deficiency

### 3.1. Overview of Human PGK1 Structure and Activity

PGK (ATP: 3-phosphoglycerate 1-phosphotransferase; EC 2.7.2.3) is a key enzyme for ATP generation in the glycolytic pathway. In humans, PGK1 has more widespread roles, particularly in oncogenesis, and tumor development and invasiveness [47]. Elevated extracellular hPGK1 concentrations turn out to inhibit plasmin-mediated angiogenesis in response to hypoxia, required for solid tumor development [48] and it was also reported that hPGK1 participates in the DNA replication and repair [49,50,51]. In addition to its physiological activity, human PGK1 displays the capability to activate L-nucleoside analogues used in anticancer and antiviral therapies [52,53,54,55,56,57].

hPGK1 is 417 amino acid long, with a molecular mass of nearly 45 kDa and a tertiary fold highly conserved among species (Figure 1). It is a typical hinge-bending enzyme with two similarly sized Rossmann fold domains [58]. The N-domain binds 1,3-BPG or 3-PG, while the C-domain binds the nucleotides. The domains are separated by a deep cleft and linked by two alpha-helices (α-helix 7 and α-helix 14) [59,60]. Contacts between the two domains are formed through hydrophobic interactions and hydrogen bonds [61,62]. The enzyme can adopt different conformational states and transition between these states can be triggered by ligand binding. Four hinge points contribute to the interdomain motions, even though, upon binding of both ligands the bending becomes restrained to a single hinge dominant point [59]. Binding of the two substrates is independent of each other and the two binding sites in the absence of ligands (open form) are rather far from each other for phosphoryl transfer. Thus, it was suggested that upon binding of the two substrates, a hinge-bending motion converts the enzyme to the closed form to allow the substrates to come into contact. The domain closure requires the concerted action of both substrates and takes place under a strong cooperativity between the two domains [60,62]. 

### 3.2. Structural and Functional Impact of Mutations in the Native hPGK1

**Figure 2:**
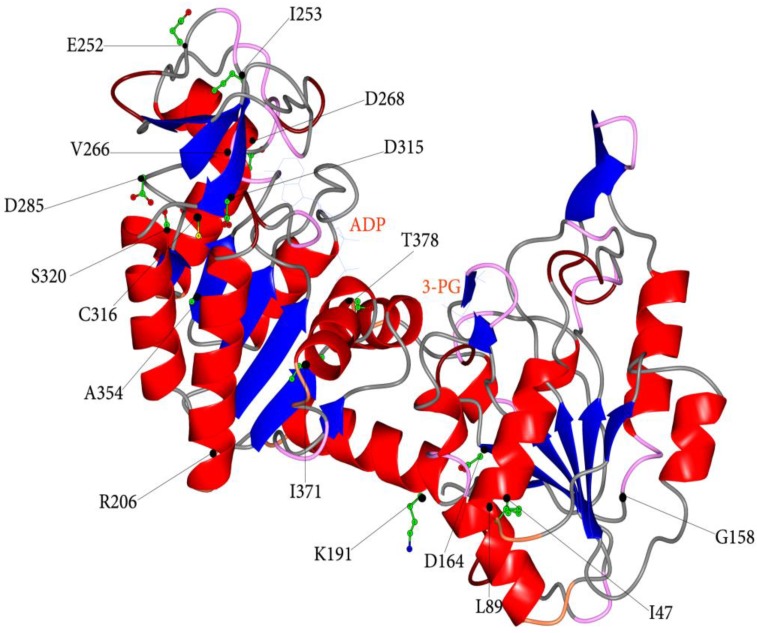
Ribbons representation of human PGK1 in open conformation (PDB ID, 2XE7). The bound ligands are shown as stick model. The side chains of amino acids affected by mutations are indicated by black spheres and represented as ball and stick.

Recently, a biochemical study addressed to provide a molecular framework to the different pathological manifestations exhibited by hPGK1 deficient patients allowed us to unravel the molecular abnormalities caused by mutations. Different clinical phenotypes correlate with the distinctive type of perturbations caused by the mutations (Table 1) [27,45]. In this study, seventeen variants of hPGK1 obtained in their recombinant form were characterized and the comparison of their molecular properties with those of the WT enzyme has enabled to determine the effects of amino acid substitutions. Localization of the amino acid residues affected by mutations is indicated in Figure 2 and summarized in Table 1. The major cause (70%) of the severe enzyme deficiency results to be the enzyme instability, even though some mutant enzymes are also affected in catalytic efficiency.

Mutant enzymes displaying highly reduced thermal stability, but moderately perturbed catalytic properties (p.I47N, p.L89P, p.C316R, p.S320N and p.A354P) show the most homogeneous correlation with hPGK1 deficient clinical phenotype (patients with hemolytic anemia, neurological disorders and, except for p.A354P, no myopathy). Based on crystallographic studies of the hPGK1 in the open and closed conformations, it is inferred that the mutations involve amino acids, which play a main role in preserving protein structure. All amino acids are part of the most common regular elements of the secondary structure (I47, L89, S320, and A354 located in α-helix 1b, 2, 11, and12, respectively; C316 in β-strand q) and participate to hydrogen/ionic interactions. In addition, I47 and L89 are involved in several hydrophobic interactions, even between them. Very interesting, L89 and A354 are substituted by a proline, a residue which is known to have a destabilizing effect on α-helical conformation and other complex effects on protein folding [63,64]. Conceivably, the highly protein instability exhibited by this group of variants leads *in vivo* to a prompt degradation of the enzyme through the ubiquitin-proteasome pathway, mainly affecting red blood cells and the central nervous system, the former being prevented from protein synthesis, the latter being unsuited to quickly replace the enzyme. Conversely, muscle cells, which rely on an efficient protein turnover, can renew the enzyme fraction damaged by a sudden increase of body temperature, as it occurs with fever or strenuous exercises. Thus, it comes that these variants, although affected in their catalytic capacity, are active enough to sustain glycolysis, providing muscle with suitable ATP production.

Another group of mutants (p.G158V, p.D164V, p.K191del, p.D285V, p.D315N, p.I371K and p.T378P) turn out to be heavily affected in both catalytic properties and protein stability, but one (p.T378P). Indeed, all amino acids affected by mutation have a role in preserving hPGK1 structure, sharing in β-strands (β-strand E, o, q and K) and α-helices (α-helix 7 and 13) and participating to several hydrogen/ionic interactions, mostly in both open and closed conformation [27,45]. Three of the residues (D315, T378 and I371) are also involved in the binding of the nucleotide, although indirectly, being located in the vicinity of this substrate binding site. For this group of variants the correlation between molecular alterations and hPGK1 deficient clinical phenotypes is less evident. With a hPGK1 enzyme so much hindered, patients should suffer from multisystem disease (all potential tissues affected). However, with the exception of the carriers of p.D164V and p.I371K, all the other patients show neither hemolytic anemia nor neurological symptoms. Curiously, in addition to carriers of p.D164V and p.I371K, three patients (carriers of p.G158V, p.D315N and p.T378P) are affected by myopathy. Therefore, it has been suggested that myopathy is in general a main trait of patients with hPGK1 enzymes displaying heavily impaired catalytic efficiency coupled to reduced protein stability.

In a third group of hPGK1 enzymes (p.R206P, p.E252A, p.I253T, p.V266M and p.D268N), the molecular properties resemble those of the WT enzyme, but intriguingly mutations are associated to heterogeneous clinical phenotypes. Thus, the genotype/phenotype relationship is quite difficult to be drawn. Very likely, amino acid substitutions are not accountable for the clinical manifestations of the carriers and the occurrence of additional genetic and/or epigenetic or other unknown factors has to be called into question for clinical phenotype explanation.

**Figure 3:**
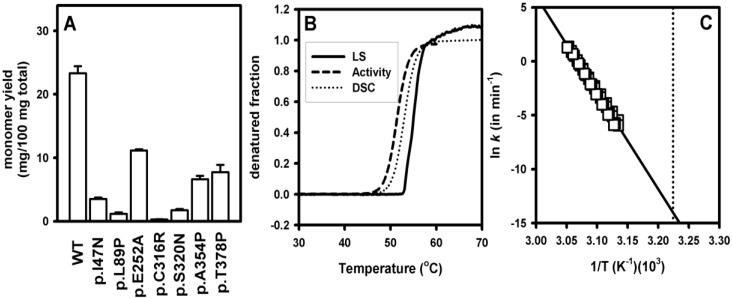
Aggregation of hPGK1 enzymes *in vitro* and upon expression in *E. coli* cultures (A) Fraction of soluble hPGK1 existing as a monomer upon expression analyses of hPGK1 enzymes in *E. coli*. The data are means ± s.d. from three independent expression experiments and were obtained starting from the same amount of total soluble protein and upon purification by ion-exchange and size exclusion chromatography as described [13,45] (**B**) Normalized thermal denaturation profiles for WT hPGK1 monitored by activity, light scattering (LS) and differential scanning calorimetry (DSC) measurements; Note that the three techniques provide similar denaturation profiles. (**C**) Arrhenius plots for the thermal denaturation of WT hPGK1 obtained from DSC experiments using a two-state irreversible model [13] and used to determine denaturation rates at 37 °C (indicated by the vertical dotted line).

**Table 1:** hPGK1 mutations, their effects on enzyme molecular properties, and clinical features in patients with hPGK1 deficiency.

Nucleotide Change	Amino Acid Change	^a ^Localization	^b ^Catalytic Properties	^c ^Protein Stability	RBC Residual Activity (%)	Hb (g/dl)	Reticulocytes (%)	^d ^Symptoms			References
								A	M	N	
c.140 T > A	^# ^p.I47N	α-helix 1b	○	○○○	8	6.6–7.3	N.A.	+	-	+	[37]
c.266 T > C	^# ^p.L89P	α-helix 2 *	○	○○○	5	N.A.	N.A.	+	-	+	[40]
c.473 G > T	^# ^p.G158V	loop α-helix 4, β-strand E	○○	○○	1	12.8	2.5	-	+	-	[65]
c.491 A > T	^# ^p.D164V	β-strand E	○○○	○○○	5	2.0–10.0	5.0-26-0	+	+/-	+	[36,38,41,66]
c.571 > 573 del	^# ^p.K191del	α-helix 7 *	○○	○○	4	14.1	6.4	-	-	-	[41]
c.617 G > C	^# ^p.R206P	loop α-helix 7, β-strand G *	-	○	10	5.6–13.7	2.0–20.0	+/-	-	+	[67]
c.755 A > C	^# ^p.E252A	loop α-helix 9, α-helix 10 *	-	-	6	13.2	N.A.	-	+	-	[68]
c.758 T > C	^# ^p.I253T	loop α-helix 9, α-helix 10 *	-	-	8	N.A.	N.A.	-	+	+	[69]
c.796 G > A c.798 C > A	^# ^p.V266M	α-helix 10a/b	-	-	10	9.3	12.5	+	-	+	[70]
c.802 G > A	^# ^p.D268N	α-helix 10b *	-	-	21	N.A.	0.4–1.3	-	-	-	[71]
c.854 A > T	^# ^p.D285V	β-strand o *	○○	○○○	49	9.0–10.0	10–45	-	-	-	[68]
c.943 G > A	^# ^p.D315N	β-strand q	○○	○○○	3	14.3	N.A.	-	+	-	[72]
c.946 T > C	^# ^p.C316R	β-strand q	○	○○○	10	7.5-13.0	1.5–5.0	+/-	-	+	[66]
c.959 G > A	^# ^p.S320N	α-helix 11	○	○○○	36	7.6	9.0	+	-	+	[73]
c.1060 G > C	^# ^p.A354P	α-helix 12 *	○	○○○	6	4.9–9.0	24	+	+	+	[37]
c.1112 T > A	^# ^p.I371K	β-strand K	○○	○○	12	12.1–14.1	4.4–5.2	+/-	+	+	[30]
c.1132 A > C	^# ^p.T378P	α-helix 13 *	○○	-	2	13.4–14.5	N.A.	-	+	-	[28,29]
IVS4+1 G > T	splicing alteration				3	N.A.	2.7	-	+	+	[74]
c.637 > 640 delGGCG	frameshift				6	N.A.	N.A.	-	+	-	[75]
c.639 C > T	splicing alteration				5	N.A.	N.A.	-	+	-	[76,77]
IVS7+5 G > A	splicing alteration				14	N.A.	N.A.	-	+	+	[78]

^# ^variants characterized; ^a ^according to [59]; * solvent accessible; ^b ^catalytic efficiency toward 3-PG or Mg-ATP: ○○○ < 1%; ○○ < 10%; ○ < 25%; - comparable to WT; ^c ^heat stability (T_m_): ○○○ nearly 10 °C lowered; ○○ nearly 3–7 °C lowered; ○ nearly 2 °C lowered; - comparable to WT; ^d ^A: anemia (+/-: compensated hemolytic anemia with occasional hemolytic crises); M: muscular disorders (+/-: sporadic manifestations); N: neurological disorders; N.A.: not available.

### 3.3. Increased Aggregation Propensity of Mutants Causing hPGK1 Deficiency: Biophysical and Expression Studies

Expression analyses in prokaryotes and eukaryotic cells are insightful tools to characterize protein misfolding in conformational diseases within a biologically relevant scenario [79,80,81,82]. To study the impact of hPGK1 mutations in the folding and aggregation propensity of hPGK1, we have expressed the WT and seven disease-causing hPGK1 enzymes in *E. coli* at 37°C. Despite this expression system oversimplifies the natural protein homeostasis environment of human proteins, it is useful to identify and characterize folding defects of disease-causing variants, and also to evaluate its potential modulation by molecular chaperones in a simple manner [80,81,82]. To determine the aggregation propensity of hPGK1 enzymes we have quantified the amount of soluble protein existing as a monomer by chromatographic methods (Figure 3A). All the hPGK1 mutants expressed in this system show a decrease in the yield of PGK monomer, ranging from 2-fold to 80-fold. This decreased folding efficiency is caused (at least partly) by an enhanced aggregation propensity, even though the impact of mutations is somewhat different on the formation of insoluble and soluble aggregates (ongoing research). These results support that hPGK1 mutants cause enzyme loss-of-function by enhancing protein misfolding, which manifests as an enhanced aggregation propensity in this expression system, but may manifest differently in eukaryotic expression systems (*i.e.*, enhanced protein turnover; [79,80,83]). 

Inactivation studies have revealed a decreased stability towards thermal induced denaturation for a large fraction of the mutants causing hPGK1 deficiency (about 75% of mutations; [13,27,45]). Decreased thermal stability, as a lower half-inactivation temperature, correlates well with a decreased kinetic stability, as a shorter half-life for irreversible inactivation [27,45]. Thermal induced inactivation of hPGK1 is caused by protein denaturation and aggregation, as shown by the similar thermal profiles found for activity, light scattering and calorimetric measurements (Figure 3B). 

We have recently performed a detailed characterization of the aggregation/denaturation kinetics of WT and disease-causing hPGK1 enzymes by differential scanning calorimetry (DSC) [13]. The presence of a single denaturation transition indicates a highly cooperative unfolding of hPGK1 for a two domain protein [13]. Thermal denaturation of hPGK1 enzymes is described well by a simple two-state irreversible conversion of the native enzyme (N) to a final state (F) that cannot fold back (N→F; [84]). This kinetic process is characterized by strongly temperature-dependent rate constant *k*, and its temperature dependence is described by the Arrhenius equation according to a given activation energy (*E*_a_). DSC analyses allow compare the rate constants between hPGK1 enzymes with very different kinetic stabilities by building up Arrhenius plots used to estimate the rate constants to a given temperature (Figure 3C). These denaturation rate constants estimated at 37°C show very different kinetic stabilities for hPGK1 enzymes, with half-lives spanning over five orders of magnitude [13], ranging from years (WT) to minutes (p.I47N and p.L89P). Interestingly, a nice correlation between the yields in monomeric hPGK1 and kinetic stabilities seems to be found (ongoing research), suggesting a link between hPGK1 kinetic stability and intracellular foldability

### 3.4. Structural and Energetic Bases of Mutation Induced Kinetic Destabilization of hPGK1

The DSC analyses on WT and mutant enzymes have also provided insight into the denaturation mechanism and the energetic bases underlying mutation induced kinetic destabilization in hPGK1 deficiency. The denaturation enthalpies of the hPGK1 mutants are strongly dependent on the individual *T*_m_ value [13]. This temperature dependence yields a denaturation heat capacity change (Δ*C*_p_) of 9.1±0.8 kcal·mol^−1^·K^−1^. This experimental Δ*C*_p_ is consistent with the global denaturation of a protein of size of hPGK1, thus indicating that irreversible denaturation of hPGK involves a large loss of tertiary structure, and also, that WT and mutant hPGK1 enzymes show similar conformations in the native and the irreversibly denatured final states [13]. Interestingly, a large dependence of *E*_a_ on *T*_m_ values was also found for this set of enzymes, which would involve a large activation Δ*C*_p_ using the same *structural* interpretation as for Δ*C*_p_, thus implying a denaturation transition state (*i.e.*, the state at the top of the kinetic free energy barrier) as *unfolded* as the irreversibly denatured state. However, we proposed an alternative interpretation to this behavior based on the *Hammond* postulate for protein folding/unfolding [85], which does not require invoking such an unstructured transition state. Accordingly, the decrease in kinetic stability (*i.e.*, in the height of the kinetic barrier) may be explained by a more native-like structure of the denaturation transition state in the most destabilizing mutants [13]. This *Hammond* behavior was experimentally supported by detailed calorimetric measurements [13], also highlighting the plasticity of the denaturation transition state in response to mutations [13] as well as to changes in environmental conditions (such as mild acidic pH, unpublished results), which agrees with the plasticity found in other proteins systems [86,87,88].

**Table 2:** Parameters describing thermodynamic and kinetic stabilities of human PGK1 WT and p.T378P enzymes. Data from [13,46].

Chemical Denaturation	WT	p.T378P
*C*_m_ (M)	2.43 ± 0.01	2.28 ± 0.06
*m*_eq_ (kcal·mol^−1^·M^−1^)	3.4 ± 0.2	1.5 ± 0.2
Δ*G*_U _(kcal·mol^−1^)	8.3 ± 0.5	3.5 ± 0.4
**Thermal denaturation**		
*T*_m_ (°C)	52.8 ± 0.2	49.8 ± 0.4
*E*_a _(kcal·mol^−1^)	191 ± 19	135 ± 12
Δ*H* (kcal·mol^−1^)	157 ± 18	127 ± 9
**Kinetic stability**		
Aggregation rate constant (*k_agg_*)(min^−1^)	9 ± 5·10^−7^	1.6 ± 0.4·10^−4^
Global unfolding rate constant (*k*_unf(0M)_) (min^−1^)	0.09 ± 0.02	0.18 ± 0.04
Proteolysis rate constant at high protease (*k*_0_) (min^−1^)	0.11 ± 0.02	0.38 ± 0.01
Proteolysis rate constant at low protease (*k*_1_) (min^−1^)	0.27 ± 0.04	7.8 ± 0.5

It must be noted that the high kinetic stability of WT hPGK1 at physiological temperature arises from a combination of a moderately high *T*_m_ (about 53 °C) and a high activation energy for irreversible thermal denaturation (of about 200 kcal·mol^−1^ for WT hPGK1; see Table 2). In an evolutionary context, we must indicate that PGK enzymes from other mesophiles (such as yeast and pig) show similarly high values of *T*_m_ and *E*_a_ [62]. An interesting possibility is that evolution may have selected some energetic features (*i.e.*, a high *E*_a_) to provide sufficient kinetic stability [13]. In the context of the human enzyme, its kinetic stability may allow the protein to operate within the life time of red blood cells (which is in the range of several months). Moreover, a highly unfolded transition state for denaturation in human, pig and yeast PGKs may also explain the large activation energy and free energy barrier for these three eukaryotic enzymes. A similar interpretation for changes in the structural properties of the irreversible denaturation transition state has been proposed for TIM proteins from different species [86,88]. Conversely, mutations causing hPGK1 deficiency lead to protein kinetic stabilization by displaying a more *native-like* denaturation transition state [13], thus showing an opposite behavior than the one evolution may have used. 

### 3.5. Dissection of the Thermodynamic and Kinetic Basis of hPGK1 Misfolding by Urea Denaturation and Proteolysis

**Figure 4:**
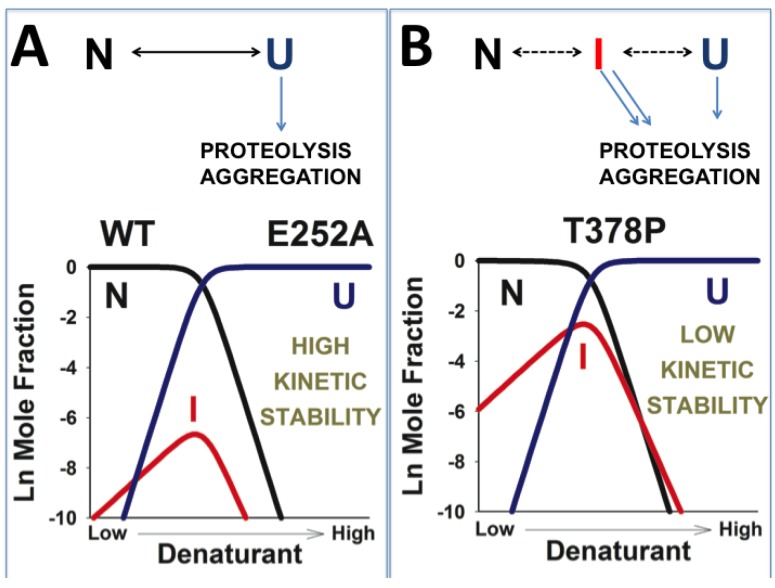
Plausible mechanisms linking the reduced kinetic stability (towards aggregation or proteolysis) and the lower unfolding cooperativity in disease-causing hPGK1 mutants. In the case of kinetically stable variants (panel **A**), the levels of the highly kinetically sensitive intermediate (I) levels are always very low, and aggregation and proteolysis occur mainly through the unfolded state U (left side of the figure). However, in some mutants (panel **B**), the population of the intermediate may raise (and can be “detected” by a low *m*-value in urea denaturations), leading to a significant contribution from the I state to the irreversible denaturation kinetics. It must be also noted that in panel B, at low denaturing stress, the I state is much more populated than the U state, and thus, may contribute much more to the kinetics of proteolysis and/or aggregation than the U state.

The phenomenological two-state model (N→F) used to describe thermal aggregation and denaturation of hPGK1 enzymes ([13] and Figure 4) does not imply that the native state undergoes the irreversible denaturation step. One of the simplest mechanisms consistent with the two-state irreversible model is the following three state mechanism: N↔U→F, in which the irreversible step is undergone from the unfolded state U. Thus, it is evident that the equilibrium between the N and U states may play a role in the kinetic stability of WT and mutant hPGK1 enzymes. Our thermal denaturation analyses support that the U state is not significantly populated, and thus, this mechanism is undistinguishable from a two state irreversible model [13]. Nevertheless, the effect of mutations in the kinetic stability of hPGK1 mutants can be further investigated by complementing our thermal denaturation studies with the use of proteolysis and equilibrium and kinetic unfolding experiments by urea, to provide a detailed picture on the relationship between thermodynamic and kinetic stabilities in hPGK1 deficiency [13,46]. We have recently applied this approach to hPGK1 WT and two thermostable mutants (p.E252A and p.T378P) which showed significant kinetic stability based on our DSC analyses [13,46], and similar analyses on less kinetically stable hPGK1 enzymes are ongoing by slightly different procedures. The main results obtained from these studies on WT and p.T378P are compiled in Table 2 and subdivided in three main sets, which are discussed in detail in the Subsections 3.5 and 3.6: (i) equilibrium denaturation parameters upon urea unfolding, *C*_m_ and *m*_eq_, which allow determine the thermodynamic stability (ΔG_U_) of the enzymes; (ii) thermal denaturation parameters, *E*_a_ (the activation energy, which determines the temperature dependence of denaturation rate constants; see Figure 3C), Δ*H* (the calorimetric enthalpy, which is the heat absorbed by the sample to be denatured) and *T*_m_ (the maximum of the thermal transition); (iii) kinetic rate constants for denaturation/aggregation (from DSC experiments), for global unfolding (from urea unfolding kinetics) and for proteolysis at low/high protease concentrations limits (from differential scanning proteolysis). The comparison between the different sets of rate constants allows to obtain information on the denaturation and proteolysis kinetic mechanisms (see below). 

Urea denaturation of WT hPGK1 shows a single transition using different structural probes [13,46] consistent with a two-state reversible unfolding model (N↔U). The free energy for unfolding determined using this model is of 8.3 kcal·mol^−1 ^for WT hPGK1, which is in the same range of the thermodynamic stability found for PGKs from *E. coli*, yeast and muscle horse [14,15]. We must note that other studies have suggested more complex mechanisms for the chemical unfolding of some PGKs possibly under conditions at which unfolding intermediates are stabilized [14,16,23]. 

There are two limiting situations in the N↔U→F mechanism proposed above [13,89]: A) the aggregation step U→F is rate-limiting, and thus the overall denaturation rate constant *k* is equal to *K*·*k*_p_; here, the kinetics of aggregation depends on the thermodynamic stability (the value of the equilibrium constant, *K*, for the N↔U step) and on the intrinsic aggregation rate (for the U→F step, *k*_p_); B). Aggregation (U→F) is faster than refolding (U→N), and thus, the overall aggregation rate constant approaches the unfolding rate constant for N→U step. Indeed, the rate constants for global unfolding determined by urea denaturation and extrapolated to the absence of denaturant show that global unfolding is much faster (3–5 orders of magnitude; Table 2) than aggregation [13,46]. This implies that the N→U step is not rate-limiting and thus, aggregation kinetics depend on both thermodynamic and kinetic factors (*K* and *k*_p_; scenario A). Equilibrium denaturation experiments also support that aggregation kinetics correlate well with a decreased thermodynamic stability of the native state, further supporting that scenario A describes well the increased aggregation propensity of hPGK1 mutants by (at least) thermodynamic destabilization of the protein [46]. 

Alternatively, we have used proteolysis to obtain further insight on the mechanism by which hPGK1 mutants reduce the kinetic stability of the native state. Kinetics of proteolysis can be studied using a similar three-state kinetic mechanism to the one proposed for aggregation kinetics hPGK1 with a small conceptual modification: the state undergoing the irreversible denaturation is a protease sensitive state (named X instead of U), while the final state F is the degraded protein. Accordingly, at low protease concentrations, proteolysis is rate-limiting (scenario A) and at high protease concentrations, proteolysis kinetics approaches to kinetics of formation of the protease sensitive state (scenario B) [90,91]. We have performed a comprehensive analysis of proteolysis kinetics at multiple temperatures and protease concentrations by differential scanning proteolysis [46,91]. Actually, we found that at high protease concentrations, the global unfolding rates of several hPGK1 enzymes are close to those of proteolysis (Table 2, [46]), supporting that the protease sensitive state *resembles* the globally unfolded state [46]. At low protease concentrations, proteolysis was 30-fold faster for p.T378P than for WT, which agrees with the reduced thermodynamic stability supported by equilibrium urea denaturation analyses (Table 2 and [46]). At high protease concentrations, proteolysis of p.T378P is 3-fold faster than for WT hPGK1, which agrees very well with the effect of this mutation on the global unfolding rate (Table 2 and [42]). 

*E. coli* PGK is 3–4 orders of magnitude more kinetically resistant towards unfolding and proteolysis than hPGK1 and yeast PGK, despite their similar thermodynamic stabilities [15,46]. This suggests in these PGK enzymes, unfolding kinetics may be driving their different proteolytic susceptibilities (at least when the proteolysis step is not rate-limiting) [15,36]. In hPGK1 and *E. coli* PGK, the kinetically sensitive state is the global unfolded state, while in yeast PGK is a partially folded state [15,36]. It has been argued that these differences may be related to the different ability of the N- and C-terminal domains of PGK enzymes to fold autonomously [15]. 

### 3.6. A Possible Role of Folding/Unfolding Cooperativity in the Loss-of-Function Mechanisms of hPGK1 Deficiency

**Figure 5:**
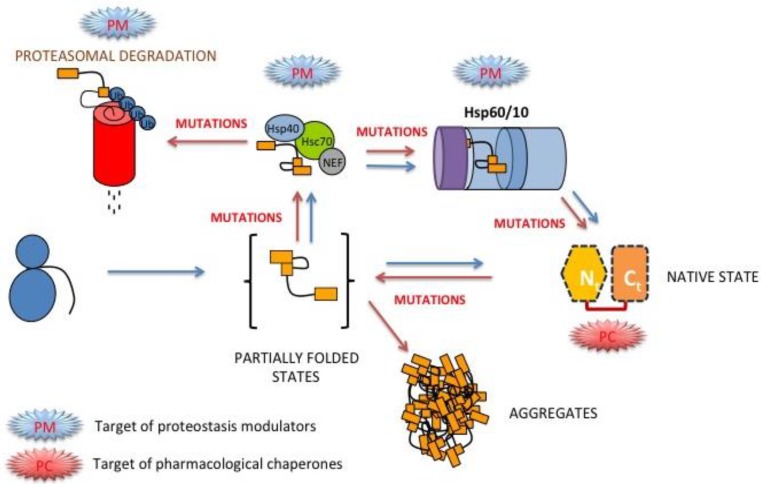
A simple scenario for protein folding and misfolding hPGK1 enzymes inside the cell. Folding of hPGK1 may occur *in vivo* spontaneously after its synthesis, as seen by its spontaneous folding *in vitro* [13] and the fast and spontaneous folding of yeast PGK in living cells ([26,99]). Alternatively, the native state can be reached via interaction of partially folded states with different classes of molecular chaperones. The red arrows indicate steps that may be affected *in vivo* upon disease-causing mutations, mostly associated with reduced native stability and folding cooperativity [13,46]. The potential targets for pharmacological correction by proteostasis modulators (PM) and pharmacological chaperones (PC) are also indicated.

It is well known that the cooperativity of the chemical denaturation of a protein following a two-state equilibrium model (*i.e.*, the equilibrium *m*-value; Table 2) scales up with protein size [92]. Indeed, urea denaturation of human, yeast and *E. coli* show remarkably similar equilibrium *m* values (of about 3.5–4 kcal·mol^−1^·M^−1^; [13,15]) consistent with their two-state behavior. However, urea denaturation of the p.T378P mutant shows much lower *m* value than for the kinetically stable WT and p.E252A enzymes, suggesting that in the p.T378P mutant, equilibrium unfolding intermediates might be populated, thus explaining the decreased folding cooperativity [13,93]. Low unfolding cooperativity is also found for the most kinetically destabilizing hPGK1 mutants (ongoing research). Thus, a scenario compatible with all these experimental results may explain the low kinetic stability of most of the hPGK1 mutants by an increased population of partially unfolded states which are sensitive to irreversible processes (such as aggregation or proteolysis) under native conditions or at low denaturing stresses (Figure 4). The population of these equilibrium intermediates must remain low for instance in thermal denaturation studies, and thus, thermal denaturation is still described well by simple models. However, the population of these intermediates, even at low levels, may be critical to determine the kinetics of irreversible denaturation [94,95,96], especially under conditions at which the unfolded state is rarely populated (Figure 4B). According to this scenario, the higher tendency to populate partially unfolded states by mutations would also imply that these states might be shielded from irreversible processes by molecular chaperones, thus providing potential pharmacological approaches to treat hPGK1 deficiency based on protein homeostasis regulators ([46] and Figure 5).

### 3.7. hPGK1 Mutants may affect Spontaneous Refolding Kinetics

As explained above, the p.T378P mutant decreases the thermodynamic stability of hPGK1 by ~5 kcal·mol^−1^, while global unfolding kinetics shows a kinetic destabilization of less than 1 kcal·mol^−1^ (Table 2; [46]). This large difference between thermodynamic and kinetic destabilization imply that this mutation may strongly affect folding kinetics, which would not be surprising because it introduces a proline residue, which are known to lead to complex effects on protein refolding kinetics [63,64]. This prompted us to study the refolding kinetics of urea denatured WT and p.T378P proteins by manual mixing techniques [46]. In contrast to urea induced unfolding kinetics, refolding kinetics showed significant complexity and some differences between these two variants were found [46]. Refolding of WT seems to occur mostly through a fast kinetic track at low denaturant concentrations, while refolding kinetics of p.T378P is slower and presents significant roll-over in the chevron plots at higher urea concentrations, which suggests the accumulation of folding kinetic intermediates in the mutant enzyme. Interestingly, alterations in folding kinetics do not seem to be specific of the p.T378P among hPGK1 disease-causing mutants (ongoing research). An interesting possibility is that the folding of disease-causing hPGK1 enzymes may require the help of molecular chaperones more stringently than the WT protein to prevent misfolding of equilibrium and kinetic intermediates ([97,98] and Figure 5).

### 3.8. Folding, Stability and Function of PGK inside the Cell

*In vitro* studies of PGK enzymes have provided remarkable information on their function, stability and folding/misfolding behavior. However, additional factors may contribute to modulate these properties inside the cell, including macromolecular crowding effects and differences in intracellular viscosity [24,99,100]. For instance, PGK enzymes have been traditionally described to perform their catalytic function through hinge-bending motions of the two domains upon substrate binding [25,101]. However, recent experimental and computational studies have demonstrated that macromolecular crowding induces a compact conformation in yeast PGK with much higher catalytic efficiency than in dilute *in vitro* conditions [25]. So, it is likely that the equilibrium between *low* and *high* activity states of PGK inside the cell is shifted to the highly active and compact conformations by macromolecular crowding effects [25]. These compact and highly active conformations have been also detected by fluorescence resonance energy transfer studies inside cells expressing doubly labeled engineered variants of yeast PGK [25]. Moreover, macromolecular crowding also seems to modulate yeast PGK stability and folding kinetics, in a cell- and compartment-specific manner [25,99]. All these studies elegantly show that PGK function, stability and folding can be tuned by several additional *physical* factors such as macromolecular crowding and viscosity. These and other *biological* factors, such as co- and post-translational folding and the interactions of partially folded states with the protein homeostasis network (chaperones, cochaperones, regulatory proteins; Figure 5) may contribute to determine the final fate of mutants associated with hPGK1 deficiency (*i.e.*, aggregation, degradation, *etc*.). Comprehensive studies of all these factors will help to understand loss-of-function in hPGK1 deficiency, as well as individual differences in the protein homeostasis network [102] which may contribute to further disclose genotype/phenotype correlations (see Section 2.2 and Section 3.2 for further details).

## 4. Perspectives for Pharmacological Intervention in hPGK1 Deficiency

Within the context of the protein homeostasis network, it is important to understand the precise molecular defects and mechanisms responsible for protein loss-of-function to design pharmacological approaches to correct mutant phenotypes. Decreased intracellular foldability of hPGK1 mutants correlate well their aggregation rates *in vitro* at physiological temperatures (ongoing research). Detailed analyses of protein stability also show that thermodynamic destabilization of the native state may play a role in aggregation/degradation of mutant enzymes (Table 2). Thus, it is plausible that development of pharmacological chaperones specifically binding to the native state would be a therapy for this disease (Figure 5). However, ligand binding to the native state of hPGK1 mutants must be understood in detail in a mutant-specific manner [13]. In this line of thinking, we have recently shown that two natural ligands of hPGK1 (the coenzymes ATP and ADP) enhance WT and mutant hPGK1 kinetic stability. However, kinetic stabilization depends on the ligand, being higher for ADP than for ATP, and on the mutant, being lower for p.T378P enzyme [13]. These differential effect may be explained by different ligand binding affinities, which is higher for ADP than for ATP, and lower for both ligands in p.T378P variant [45]. So, we must take into account the ligand binding affinity and energetics in order to find suitable pharmacological ligands for hPGK1 deficiency. Beyond the native state, we must also consider that the most kinetically destabilizing variants also show more *structured* transition states [13], and thus, we should also consider the possibility that some native state ligands would also bind to the denaturation transition state [103], and thus, the kinetic stabilization would be lower (or even negligible). 

Molecular chaperones are known to help proteins to fold and to prevent aggregation of partially folded states [4]. Our unfolding studies with hPGK1 show that the WT enzyme unfolds reversibly by urea in an apparent two state fashion, supporting that no equilibrium intermediates are significantly populated *in vitro* [13]. However, hPGK1 disease-causing variants often reduce thermodynamic stability and unfolding cooperativity ([46] and ongoing research), which may imply a higher population of unfolding intermediates *in vitro* (and possibly intracellularly). Interestingly, the p.T378P mutant also seems to slow down refolding kinetics [46]. The higher population of kinetic/equilibrium intermediates in disease-causing hPGK1 mutants could also explain the high aggregation propensity at physiological temperatures in hPGK1 disease-causing mutants both *in vitro* and in *E. coli* expression studies. Thus, it is reasonable that targeting the coordinated action of molecular chaperones on hPGK1 folding intermediates may also allow increase the protein folding efficiency of these disease causing mutants (Figure 5). Phenotypic correction by pharmacological modulation of chaperone networks has been described for several conformational diseases [8,104]. However, due to the large complexity of these networks (comprising over 800 proteins; [4]), we must first identify those targets within the protein homeostasis network involved in the folding and misfolding of disease-causing hPGK1 variants (ongoing research). 
